# A Case Presentation of Well-Differentiated Hepatocellular Carcinoma With No Sign of Liver Disease

**DOI:** 10.7759/cureus.61635

**Published:** 2024-06-04

**Authors:** Prachi Gedekar, Atul Chavhan, K. M. Hiwale, Shakti Sagar

**Affiliations:** 1 Medicine, Datta Meghe Institute of Higher Education and Research, Wardha, IND; 2 Pathology, Datta Meghe Institute of Higher Education and Research, Wardha, IND

**Keywords:** mature hepatocytes, histopathology (hp), computed tomography (ct ), hepatocellular carcinoma (hcc), liver biopsy

## Abstract

The type of liver cancer that occurs most frequently is hepatocellular carcinoma (HCC). The majority of cases of HCC are secondary to alcoholic cirrhosis or viral hepatitis. The presence of malignant cells with modest nuclear atypia that resemble normal hepatocytes and the lack of bare nuclei in the smears, which shows the neoplastic hepatocytes' capacity, are characteristics of a well-differentiated HCC plasma membrane to tolerate smearing. We present the case of an 83-year-old male patient with a well-differentiated HCC, who had no etiological factors and no signs of alcohol cirrhotic liver, or any symptoms of liver disease which are the main causes of the HCC.

## Introduction

Globally, liver cancer has the fourth position in cancer-related fatalities and is the fifth most prevalent type of cancer. It is the fourth most common malignancy and the second most common cause of cancer-related death among men worldwide [1]. Compared to women, men are expected to acquire liver cancer [1]. Intrahepatic cholangiocarcinoma (ICC) and hepatocellular carcinoma (HCC) are two common forms of primary liver cancer; angiosarcoma, hemangiosarcoma, and hepatoblastoma are unusual forms [1]. Aristolochic acid and aflatoxins in food, chronic hepatitis B and C, alcoholism, and metabolic liver disease, especially nonalcoholic fatty liver disease are adverse conditions for HCC. Conventionally, histology or cytology has been used to make the diagnosis of HCC [2].

Early-stage HCC is asymptomatic, which causes a considerable delay in receiving a quick diagnosis. Patients with advanced HCC have limited access to effective therapy alternatives, and those with a diagnosis at the final stage of the disease are not eligible for curative surgery. The most popular biomarker for HCC surveillance and diagnosis is alpha-fetoprotein (AFP). It is utilized in conjunction with multiphasic computed tomography (CT), magnetic resonance imaging (MRI), and ultrasound scanning (USS) as the sole phase V biomarker currently available for HCC surveillance [3]. One of the characteristics of cancer is metabolic change. Lipid accumulation is a distinctive pathological characteristic of HCC, occurring frequently in a well-differentiated HCC and infrequently in poorly differentiated HCC. It is common that depending on the grade of differentiation, HCC might alter the pathological characteristics. Of these, a well-differentiated HCC is recognized for being a fat-containing tumor; as the degree of differentiation decreases to moderate or poorly differentiated, the stored fat vanishes. It is believed that inadequate arterial development and reduced portal vein flow are the root causes of this fatty alteration in early-stage HCC [4]. Hepatocellular adenoma in non-cirrhotic liver and high-grade dysplastic nodule in cirrhosis can be mimicked by a well-differentiated HCC [5]. HCCs are thought to expand and become more malignant over time, progressing progressively from a well-differentiated HCC to intermediate HCC and then poorly differentiated HCC. Therefore, well-differentiated HCCs, whose average diameter is less than 20 mm, are an early form of HCCs in hepatocarcinogenesis. The portal vein and hepatic artery supply the HCC and the liver parenchyma. Generally speaking, well-differentiated HCCs develop into moderately differentiated HCCs when the tumor's primary blood supply shifts from the portal tracts to the arteries. Diverse fatty tissue grades are frequently present in HCCs. This fatty transformation is more common in tiny, well-differentiated HCC nodules, particularly those with a diameter of less than 20 mm. A well-differentiated HCC with fatty alteration is quite uncommon to progress to over 30 mm [6].

## Case presentation

**Figure 1:**
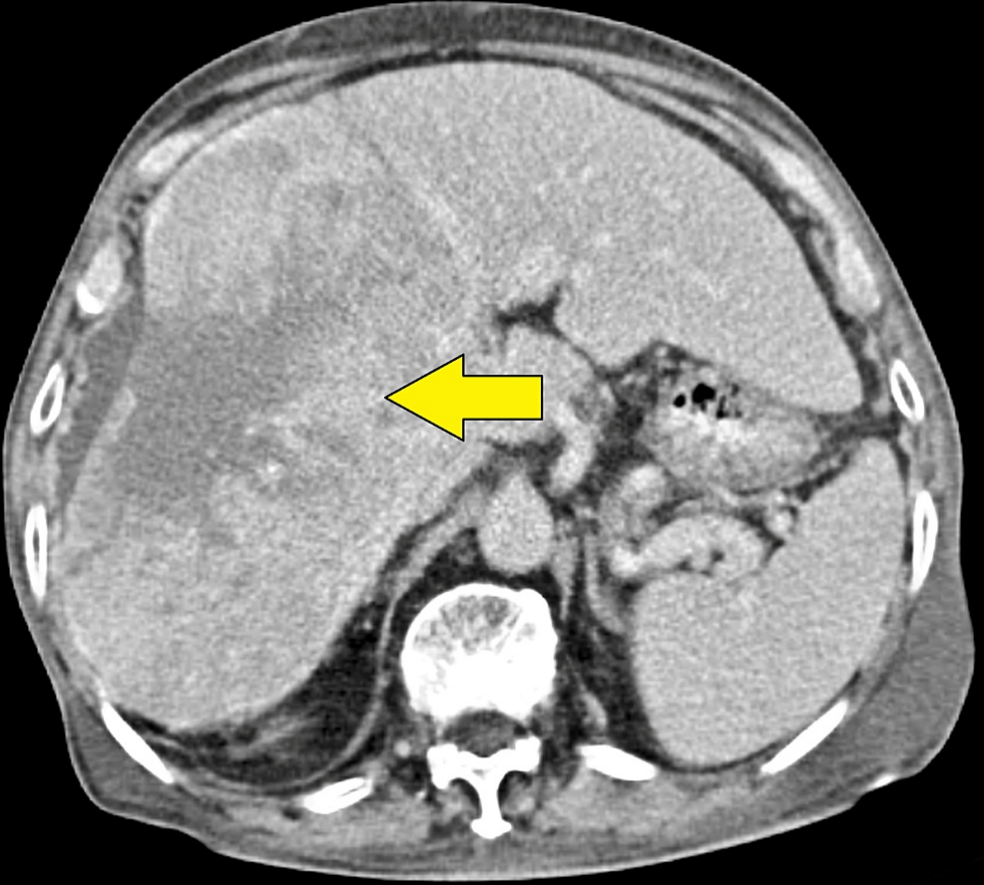
Computed tomography revealed a hypoechoic lesion in the right lobe of the liver (indicated by yellow arrow) with ascites

We present the case of an 83-year-old man who complained of stomach pain and was admitted to the hospital. He also reported low appetite and swelling on his limb. Large heterogeneously enhancing lesions are visible on the CT image of the abdomen and pelvis, and a well-circumscribed soft tissue density mass lesion measuring 14 × 13 cm is observed in the right lobe of the liver (Figure 1). The mass injury is causing capsular retraction with surrounding perihepatic fluid. The examination of the abdomen is completed. Examination of other systems was unremarkable.

 A lab investigation was done. All lab parameters are normal as shown in Table 1.

**Table 1:** Lab results and reference ranges for the patient

INVESTIGATION	OBSERVED VALUE	UNIT	REFERENCE RANGE
Hemoglobin Hb%	12	Gm%	Male 13-17; female 12-15 gm%
Red blood cell (RBC) count	3.62	Millions/cu.mm	Male 4.5-5.5 millions/cu.mm; female 3.8-4.8 millions/cu.mm
Whole blood count (WBC)	7300	/cu.mm	Male and female 4000-10000 cu.mm
Total platelet count	187000	100000/cu.mm	Male and female 150000-410000 Lacs/cu.mm
Prothrombin time (PT)	11	Second	10-12 sec
Partial thromboplastin time (PTT)	31	Second	25-38 sec
Serum alpha-fetoprotein	13	Ng/ml	<7.22 IU/ml

**Figure 2:**
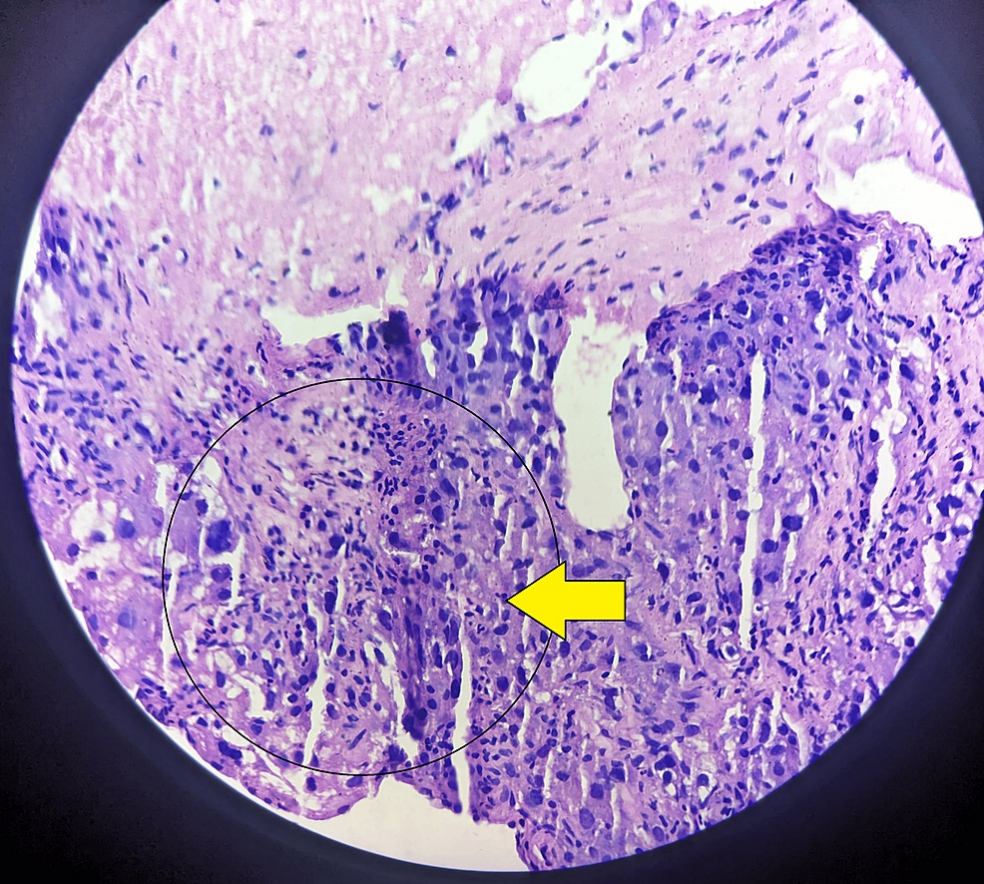
Microscopy of the liver biopsy shows tumor cells in trabecullar pattern with mature hepatocytes with eosinophilic cytoplasm

Multiple core biopsies, collectively measuring 1.0 × 0.2 cm, were received at the histopathology laboratory. The histopathology report revealed that sections show hyperchromatic nuclei tumor cells arranged in a trabecular pattern, resemble large round to polygonal mature hepatocytes, large pleomorphic nuclei, mild nuclear atypia, and clear to eosinophilic cytoplasm well-differentiated HCC as shown in Figure 2.

Medications given to the patient during hospitalization are shown in Table 2.

**Table 2:** Medicine given in the hospital OD: Once a day (omne in die); BD: twice daily (bis in die)

#	Medicine	Quantity	Doses	Days
1	Tablet multivitamin	10	OD	10
2	Tablet vitamin C	10	OD	10
3	Tablet pantoprazole	10	OD	10
4	Tablet acetaminophen	10	BD	10
5	IV fluid vitamin K in 500 ml NS	1	OD	1

After the reports of histopathology, the disease was explained to the patient, and the patient was advised to stay and be treated in the hospital, but the patient wants to be discharged from the hospital and thus has been given discharge upon patient request with prescription shown in Table 3.

**Table 3:** Medicine given to the patient on discharge

#	Medicine	Quantity	Doses	Days
1	Tablet multivitamin	10	OD	10
2	Tablet vitamin C	10	OD	10

## Discussion

Thompson et al.'s study demonstrates that compared to weakly differentiated HCC, around 28% of HCC tumors with well and moderate differentiation are more rigid, implying that tumor stiffness as measured by magnetic resonance elastography (MRE) might be a helpful imaging biomarker to distinguish between different HCC tumor grades [7]. It is unclear how highly differentiated HCC differs from poorly differentiated HCC in terms of stiffness. Decreased vascularity and greater necrosis could account for the reduced stiffness in poorly differentiated HCC [7]. However, the part of tumor stiffness only took into account the solid, enhancing portion of the tumor estimate in the current investigation. One theory holds that by definition, highly and moderately differentiated HCCs exhibit an extra-organized tissue architecture trabecular pattern in contrast to weakly differentiated HCCs, which retain their trabecular pattern HCC [7].

As per the study of Hong et al., aberrant reticulin stain, pattern-reduced reticulin stain, or expanded trabeculae are thought to be accurate in identifying a well-differentiated HCC, while odd reticulin stain patterns can occasionally be found. A portion of HCC's maintained reticulin staining represents a variety of tumor reticulin staining patterns reported in this article. In tiny samples like cellblocks or small core biopsies, an intact reticulin staining pattern is uncommon, but it has to be identified when making a diagnosis of a well-differentiated HCC [8].

Kim's study demonstrates that in most well-differentiated HCCs, improved by superparamagnetic iron oxide (SPIO), 85% had hyperintensity; however, these tumors also displayed varied CT density patterns. The majority of hyperintense, well-differentiated HCCs as shown on SPIO-enhanced MRI were influenced by the CT density pattern [9].

## Conclusions

An 83-year-old male presented with a massive well-differentiated hepatocyte carcinoma (HCC) in the right lobe of the liver, although there were no distant lesions. Hepatocellular carcinoma typically develops in the context of chronic cirrhosis related to hepatitis B and hepatitis C infections. This case involves an 83-year-old male patient with a well-differentiated HCC in a non-cirrhotic liver, despite the absence of chronic hepatitis or any evident etiological or risk factor for HCC.
